# Sleep quality in Olympic and non-Olympic combat sports: associations with sex, rapid weight loss, and sport age (a PSQI-based analysis)

**DOI:** 10.3389/fpsyg.2026.1847782

**Published:** 2026-06-03

**Authors:** Şeref Eroğlu, Tülay Ceylan, Mehmet Türkmen, Taner Yılmaz, Osman Imamoğlu, Irfan Kara, Gülşah Sekban, Fatma Neşe Sahin

**Affiliations:** 1 Ministry of Youth and Sports, Ankara, Türkiye; 2 Department of Coaching Education, Faculty of Sport Sciences, Istanbul Esenyurt University, Istanbul, Türkiye; 3 Department of Physical Education and Sport, Faculty of Yaşar Doğu Sport Sciences, Ondokuz Mayıs University, Samsun, Türkiye; 4 Department of Physical Education and Sport, Faculty of Sport Sciences, Uşak University, Uşak, Türkiye; 5 Department of Sport Management, Faculty of Yaşar Doğu Sport Sciences, Ondokuz Mayıs University, Samsun, Türkiye; 6 Department of Exercise and Sport Sciences for the Disabled, Faculty of Sport Sciences, Istanbul Gelişim University, Istanbul, Türkiye; 7 Department of Physical Education and Sports, Faculty of Sport Sciences, Sinop University, Sinop, Türkiye; 8 Department of Coaching Education, Faculty of Sport Sciences, Ankara University, Ankara, Türkiye

**Keywords:** combat sports, rapid weight loss, sex differences, sleep disturbance, sleep quality

## Abstract

**Background:**

Sleep quality is an important component of recovery and performance in athletes. Combat sport athletes may be at increased risk of sleep disturbances due to high training demands and weight-management practices.

**Methods:**

This cross-sectional study included 498 athletes competing in Olympic and non-Olympic combat sports (mean age ≈21 years). Poor sleep quality (PSQI > 5) was examined using multivariable logistic regression to assess associations with sex, sport type, rapid weight loss (>4%), and sport age. A secondary model was conducted in the Olympic subsample. Continuous sleep outcomes were analyzed using two-way ANOVA.

**Results:**

Poor sleep quality was common among athletes, with 65% of participants classified as poor sleepers (PSQI > 5). In the full sample, male sex (OR = 1.65, 95% CI: 1.20–2.30), Olympic sport participation (OR = 1.75, 95% CI: 1.25–2.45), and rapid weight loss >4% (OR = 2.70, 95% CI: 1.80–4.00) were associated with higher odds of poor sleep quality, and sport age was also positively associated (OR = 1.03, 95% CI: 1.01–1.05). Similar associations were observed in the Olympic subsample. In addition, athletes competing in Olympic combat sports showed higher scores across several PSQI components compared to those in non-Olympic disciplines.

**Conclusion:**

Poor sleep quality is common among combat sport athletes and is associated with sport type and weight-management practices. These findings suggest that sleep disturbances may be related to sport-specific demands. Further research using longitudinal designs and objective sleep measures is needed to clarify these associations.

## 1 Introduction

Healthy sleep is essential for maintaining cognitive functioning, physiological balance, and psychological well-being. Disruptions in sleep quality and duration have been shown to impair learning, attention, and decision-making, while also leading to adverse effects on metabolic and immune systems (Irwin, 2015; Mukherjee et al., 2024). Insufficient sleep has been associated with an increased risk of obesity and cardiovascular diseases and may also impair mood regulation by exacerbating chronic stress responses (Wang and Boros, 2021; Walsh et al., 2021). Several factors, including stress levels, irregular living conditions, environmental stimuli, and the nature of physical load, have been identified as key contributors to sleep disturbances (Chandrasekaran and Fernandes, 2020; Güney and Imamoglu, 2022). Overall, these findings highlight that sleep quality is not only vital for general health but also represents a critical factor for athlete populations exposed to high performance and recovery demands.

Research involving athletes suggests that poor sleep quality is a common issue, with prevalence rates ranging from 38% to 57% (Gupta et al., 2017). Insufficient sleep may increase injury risk due to elevated fatigue and can impair recovery by disrupting hormonal and metabolic processes (West, 2018). When inadequate sleep persists alongside high training loads, it has been linked to suppressed immune function and reduced cognitive performance (Kirschen et al., 2020). These effects may negatively influence not only physical performance but also cognitive functions such as attention, decision-making, and academic functioning (Curcio et al., 2006; Lim et al., 2021). In addition, research conducted on adolescent wrestlers has demonstrated that regular wrestling training is closely associated with psychological well-being, reduced anxiety levels, and improved resilience, highlighting the strong interaction between training load, mental health, and overall recovery processes (Özkan et al., 2026).

Sleep quality in athletes is widely recognized as a key health indicator closely linked to performance outcomes and recovery capacity (Akillioglu and Cherkashin, 2026). Among athletes exposed to high physical and psychological demands, insufficient sleep has been associated with performance decline, increased injury risk, and physiological imbalances. In combat sports, athletes may also engage in rapid weight loss practices prior to competition due to weight-category requirements, often reducing a notable proportion of body mass within a short period through methods such as dehydration, energy restriction, and intensive training loads (Amawi et al., 2024; Wilbraham et al., 2024; Çaglar et al., 2025). These practices have been associated with adverse effects on cardiovascular function, muscle strength, and the immune system, and may also contribute to disturbances in mood regulation and impairments in sleep quality (Lakicevic et al., 2024; Sariakçali et al., 2025). In contrast, athletes competing in non-Olympic combat sports are exposed to similar training and competitive demands but may differ in the structure and regulation of weight-management practices (Reale et al., 2017).

Despite the growing body of research on sleep quality in athletes, existing studies have largely focused on single combat sport disciplines or have not clearly distinguished between Olympic and non-Olympic contexts. This distinction may be relevant, as Olympic combat sports are generally characterized by more standardized competition structures, formalized qualification systems, and regulated weight-category requirements. However, this classification should not be interpreted as a direct indicator of competitive level, as athletes in non-Olympic combat sports may also compete at high-performance levels and be exposed to substantial training and competitive demands. In addition, sex-related differences in sleep quality have often been examined independently of sport type, and the joint contribution of sex and sport context to sleep outcomes remains insufficiently explored. Furthermore, although rapid weight loss is a common behavior in combat sports, its potential contribution to sleep quality in this context is not fully understood. Given that rapid weight loss was assessed retrospectively based on self-reported pre-competition weight change, it was considered a secondary exploratory variable in the present study. Therefore, a clear research gap exists regarding how sport type (Olympic vs. non-Olympic) and sex are jointly associated with sleep quality in combat sport athletes, as these factors may interact to influence both the prevalence and severity of sleep disturbances in different ways.

### 1.1 Hypotheses

H1: Athletes competing in Olympic combat sports would demonstrate poorer sleep quality profiles compared to those in non-Olympic combat sports.

H2: Sleep quality would differ according to sex in combat sport athletes.

H3: Rapid weight loss greater than 4% would be examined as a secondary exploratory variable in relation to poor sleep quality.

H4: Sport age would be positively associated with poor sleep quality.

## 2 Materials and methods

### 2.1 Research design

This study employed a cross-sectional quantitative design with a comparative approach. The primary aim was to compare sleep quality between athletes competing in Olympic and non-Olympic combat sports with respect to sex. In addition, rapid weight loss and sport age were examined as supplementary variables potentially associated with poor sleep quality. Data were collected between January and April 2025 using an online questionnaire. The survey was distributed through sports clubs and national team communication channels, and participants voluntarily completed the questionnaire after providing informed consent.

To further explore factors associated with poor sleep quality, a secondary multivariable logistic regression analysis was conducted in the Olympic combat sports subsample. In this model, poor sleep quality (PSQI > 5) was included as the dependent variable, while sex, weight loss category ( ≤ 4% vs. >4%), and sport age were entered as independent variables. Rapid weight loss was included as an additional variable due to its relevance in weight-category sports.

### 2.2 Participants

Athletes actively competing in combat sports and regularly participating in structured training programs and official competitions were included in the study. A total of 498 competitive combat sport athletes were recruited from organized competitive sport settings, including licensed sports clubs and national-level training camps. Recruitment was not intended to represent different competitive levels across Olympic and non-Olympic groups; rather, both groups consisted of athletes actively engaged in structured training and official competition.

Data were collected within a short period following the most recent competition (typically within 1–4 weeks), and participants were asked to retrospectively report their body weight changes during the final 7 days prior to that competition. This retrospective approach may introduce recall bias and should be considered when interpreting weight-related variables.

This criterion was applied to both Olympic and non-Olympic combat sport athletes to reduce potential differences in competitive background between groups. Participants were classified according to sport type based on their inclusion in the official Olympic Games program, specifically according to the current Olympic program (e.g., Paris 2024 cycle). The classification into Olympic and non-Olympic combat sports was based on inclusion in the official Olympic Games program and was intended to reflect differences in sport structure (e.g., competition format, qualification systems, and weight-category regulations), rather than to indicate differences in athlete performance level. To further reduce potential differences in competitive level between groups, all participants were required to have recent experience in national or international competitions and to be actively engaged in structured training programs. Therefore, both Olympic and non-Olympic groups consisted of competitive athletes rather than recreational or purely local-level participants. Olympic combat sports included boxing (*n* = 65), wrestling (*n* = 70), judo (*n* = 55), and taekwondo (*n* = 60), whereas non-Olympic combat sports included karate (*n* = 80), kickboxing (*n* = 95), and Muay Thai (*n* = 73). A total of 498 athletes (male = 259; female = 239) participated in the study, with a balanced representation between Olympic (*n* = 250) and non-Olympic (*n* = 248) disciplines. A stratified sampling approach was employed to ensure proportional gender distribution across all combat sport categories.

Participants were excluded if they reported diagnosed sleep disorders, medical conditions affecting sleep, or incomplete questionnaire responses. Detailed information regarding the distribution of participants by sex and sport discipline is presented in Table 1.

**Table 1:** Descriptive characteristics of participants.

Variable	Total (*n* = 498)	Male (*n* = 259)	Female (*n* = 239)	Olympic (*n* = 250)	Non-Olympic (*n* = 248)
Age (years)	21.3 ± 2.4	21.5 ± 2.5	21.0 ± 2.3	21.4 ± 2.5	21.2 ± 2.3
Sport age (years)	9.4 ± 4.8	10.2 ± 5.0	8.5 ± 4.5	10.0 ± 4.9	8.8 ± 4.6
Height (m)	1.68 ± 0.08	1.73 ± 0.07	1.62 ± 0.06	1.69 ± 0.08	1.67 ± 0.08
Weight (kg)	65.4 ± 10.8	71.2 ± 10.5	58.8 ± 8.5	66.5 ± 10.5	64.3 ± 11.0
BMI (kg/m^2^)	23.1 ± 3.3	23.8 ± 3.4	22.4 ± 3.1	23.3 ± 3.2	22.9 ± 3.4
Rapid weight loss >4% *n* (%)	210 (42%)	125 (48%)	85 (35%)	140 (56%)	70 (28%)
Poor sleep quality (PSQI >5) *n* (%)	325 (65%)	185 (71%)	140 (58%)	185 (74%)	140 (56%)

### 2.3 Study procedure

Eligible athletes were informed about the study and provided written informed consent before participation. Data were collected under standardized conditions prior to training sessions in training environments where the athletes were actively engaged (e.g., sports clubs and national team training camps). Participants completed the Pittsburgh Sleep Quality Index (PSQI) and reported their sex, sport discipline, sport age, and pre-competition weight-loss practices. Weight loss percentage was calculated from self-reported body weight change during the 7 days preceding the most recent competition.

### 2.4 Outcome measures

#### 2.4.1 Sleep assessment

Sleep quality was assessed using the Pittsburgh Sleep Quality Index (PSQI), a widely used self-report instrument for evaluating sleep quality over the previous month (Buysse et al., 1989; Mollayeva et al., 2016). Higher scores indicate poorer sleep quality. Consistent with established criteria, a PSQI score >5 was used to define poor sleep quality (Agargün et al., 1996).

The PSQI was administered prior to training sessions under comparable conditions to minimize the potential influence of acute training effects. In the present study, the internal consistency of the PSQI was acceptable (Cronbach's alpha = 0.82).

#### 2.4.2 Rapid weight loss

Rapid weight loss (RWL) was assessed based on self-reported body weight changes during the seven days preceding the most recent competition, a time frame commonly associated with pre-competition weight-cutting practices in combat sports. Data were collected within a short period following the most recent competition (typically within 1–4 weeks), and participants were asked to retrospectively report their body weight changes during the final 7 days prior to that competition. The assessment did not distinguish between different weight-cutting strategies (e.g., gradual dietary restriction vs. acute dehydration within the final 24–48 h), and therefore reflects the overall magnitude of weight reduction rather than the specific method used.

Participants were asked to report:

(i) their highest body weight during the final week of preparation, representing their typical training weight within that period, and
(ii) their official pre-competition weigh-in body weight.

The percentage of body weight loss was calculated using the following formula:


$$\begin{array}{l} \left(\text{“highest body weigh}{\text{t}}^{\prime \prime }-\text{“pre}-\text{competition weigh}{\text{t}}^{\prime \prime }\right)/\\ \text{“highest body weigh}{\text{t}}^{\prime \prime }\times 100 \end{array}$$

Athletes were subsequently categorized into two groups ( ≤ 4% and >4%), with weight loss exceeding 4% classified as rapid weight loss, consistent with previous literature in combat sports (Lakicevic et al., 2020; Reale et al., 2018).

All weight-related data were obtained via self-report and were not independently verified. Given that RWL was assessed retrospectively using self-reported data, findings related to this variable should be interpreted with caution.

### 2.5 Statistical analysis

The primary outcome of the study was poor sleep quality, defined as a Pittsburgh Sleep Quality Index (PSQI) total score >5. To identify independent predictors of poor sleep quality, a multivariable logistic regression model was performed in the full sample (*n* = 498). Sex, sport type (Olympic vs. non-Olympic combat sports), rapid weight loss category ( ≤ 4% vs. >4%), and sport age were entered as independent variables. In addition, the sex × sport type interaction term was included to explore potential differential effects across groups. In line with the study design, rapid weight loss was treated as a secondary exploratory variable rather than a primary explanatory factor. This model was considered the main analytical framework for evaluating the overall correlates of poor sleep quality.

As a secondary analysis, a separate multivariable logistic regression model was conducted in the Olympic combat sports subsample (initial *n* = 250) representing the subset of athletes classified as competing in Olympic combat sports based on the study classification criteria. This model included sex, rapid weight loss category, and sport age as predictors. The purpose of this analysis was to examine whether the observed associations remained consistent within a more homogeneous competitive context. In addition to the categorical outcome, continuous sleep parameters (PSQI total score and component scores) were analyzed as secondary outcomes. To examine group differences, two-way analyses of variance (ANOVA) were conducted in the full sample with sex and sport type as fixed factors. A separate two-way ANOVA was also performed within the Olympic subsample using sex and rapid weight loss category as factors. When significant interaction effects were detected, pre-specified simple effects analyses were performed. Due to missing data in one or more variables included in the regression model, four participants from the Olympic subsample were excluded from the multivariable analysis, resulting in a final analytic sample of *n* = 246.

All statistical analyses were performed using IBM SPSS Statistics for Windows, Version 20.0 (IBM Corp., Armonk, NY, USA). Statistical significance was set at *p* < 0.05. Prior to parametric analyses, assumptions of normality and homogeneity of variance were evaluated using visual inspection and standard diagnostic approaches. Given the relatively large sample size, parametric tests were considered robust to minor deviations from normality. To control for Type I error across multiple comparisons involving PSQI components, the Holm–Bonferroni correction was applied. Effect sizes were reported to facilitate interpretation of practical significance. For logistic regression models, results were expressed as odds ratios (ORs) with 95% confidence intervals (CIs). For ANOVA, partial eta squared (η*p*^2^) was reported, and for pairwise comparisons, Hedges' *g* with 95% CIs was calculated and interpreted using standard thresholds (small, moderate, large). Model performance for logistic regression analyses was evaluated using the Hosmer–Lemeshow goodness-of-fit test, Nagelkerke *R*^2^, and the area under the receiver operating characteristic curve (AUC) to assess discrimination ability. Multicollinearity among predictors was examined using standard diagnostic criteria and was not considered problematic.

To ensure the robustness of the logistic regression models, additional diagnostic checks were performed, including assessment of multicollinearity using variance inflation factors (VIF), evaluation of model fit using the Hosmer–Lemeshow test, and discrimination using the area under the ROC curve (AUC). The sample size was considered adequate based on established events-per-variable criteria. No evidence of multicollinearity was observed among predictors. Model fit was acceptable according to the Hosmer–Lemeshow test, and discrimination ability was supported by the AUC value. The sex × sport type interaction term was retained based on its theoretical relevance, even though it did not reach statistical significance, to allow for a more comprehensive evaluation of potential group-specific patterns.

All statistical analyses were performed using IBM SPSS Statistics for Windows, Version 20.0 (IBM Corp., Armonk, NY, USA). Data were analyzed using. Statistical significance was set at *p* < 0.05.

## 3 Results

The results are presented in the following sections, including descriptive statistics, primary regression analysis, and secondary analyses.

Descriptive characteristics of the participants are presented in Table 1.

Participants had a mean age of 21.3 years ± 2.4 years, with male athletes being slightly older and having greater sport age compared to female athletes (Table 1). Mean height and body mass were 1.68 m ± 0.08 m and 65.4 kg ± 10.8 kg, respectively, with higher values observed in male athletes (1.73 m ± 0.07 m; 71.2 kg ± 10.5 kg) than in female athletes (1.62 m ± 0.06 m; 58.8 kg ± 8.5 kg). BMI values were similar across groups, although slightly higher in males (23.8 kg/m^2^ ± 3.4 kg/m^2^) compared to females (22.4 kg/m^2^ ± 3.1 kg/m^2^).

Rapid weight loss (>4%) was reported by 42% of the total sample (210/498), with a higher prevalence in male athletes (48%) compared to female athletes (35%). When stratified by sport type, a markedly higher proportion of athletes in Olympic combat sports reported rapid weight loss (56%) compared to those in non-Olympic disciplines (28%).

Poor sleep quality (PSQI > 5) was observed in 65% of participants (325/498). The prevalence of poor sleep quality was higher in male athletes (185/259, 71%) than in female athletes (140/239, 58%). Similarly, athletes competing in Olympic combat sports showed a higher prevalence of poor sleep quality (74%) compared to those in non-Olympic combat sports (56%).

Table 2 presents the results of the multivariable logistic regression analysis for poor sleep quality (PSQI > 5) in the full sample. Male sex, Olympic sport participation, rapid weight loss >4%, and sport age were associated with higher odds of poor sleep quality. The magnitude of associations varied across predictors, although rapid weight loss remained among the variables associated with increased odds. The interaction between sex and sport type was not statistically significant.

**Table 2:** Multivariable logistic regression analysis of poor sleep quality (PSQI > 5) in the full sample (*n* = 498).

Variable	OR	95% CI	*p*
Male sex	1.65	1.20–2.30	0.002
Olympic sport	1.75	1.25–2.45	0.001
>4% weight loss	2.70	1.80–4.00	< 0.001
Sport age (years)	1.03	1.01–1.05	0.020
Sex × Sport type	1.20	0.90–1.60	0.120

Table 3 presents the results of the multivariable logistic regression analysis for poor sleep quality (PSQI > 5) among athletes competing in Olympic combat sports. Male sex and rapid weight loss >4% were associated with higher odds of poor sleep quality. Sport age showed a modest positive association with poor sleep quality. The model demonstrated acceptable fit (Hosmer–Lemeshow *p* = 0.41) and adequate discrimination (AUC = 0.72), with modest explanatory power (Nagelkerke *R*^2^ = 0.18).

**Table 3:** Multivariable logistic regression model for predictors of poor sleep quality (PSQI > 5) in Olympic combat sport athletes (*n* = 246).

Variable	OR	95% CI	*p*
Sex (reference: female)			
Male	1.82	1.28–2.58	0.001
Weight loss (reference: ≤ 4%)			
>4% weight loss	2.41	1.53–3.79	< 0.001
Sport age (years)	1.04	1.01–1.07	0.021

Model fitted in athletes competing in Olympic combat sports only (*n* = 246). Odds ratios (ORs) are adjusted for all variables included in the model. The model showed acceptable fit (Hosmer–Lemeshow *p* = 0.41), adequate discrimination (AUC = 0.72, 95% CI: 0.66–0.78), and modest explanatory power (Nagelkerke *R*^2^ = 0.18). Events/non-events = 165/81.

Significant interaction effects between sex and sport type were identified for several sleep outcomes, including subjective sleep quality, habitual sleep efficiency, sleep medication use, daytime dysfunction, and the total PSQI score, indicating that the association between sport type and sleep outcomes differed according to sex.

**Figure 1:**
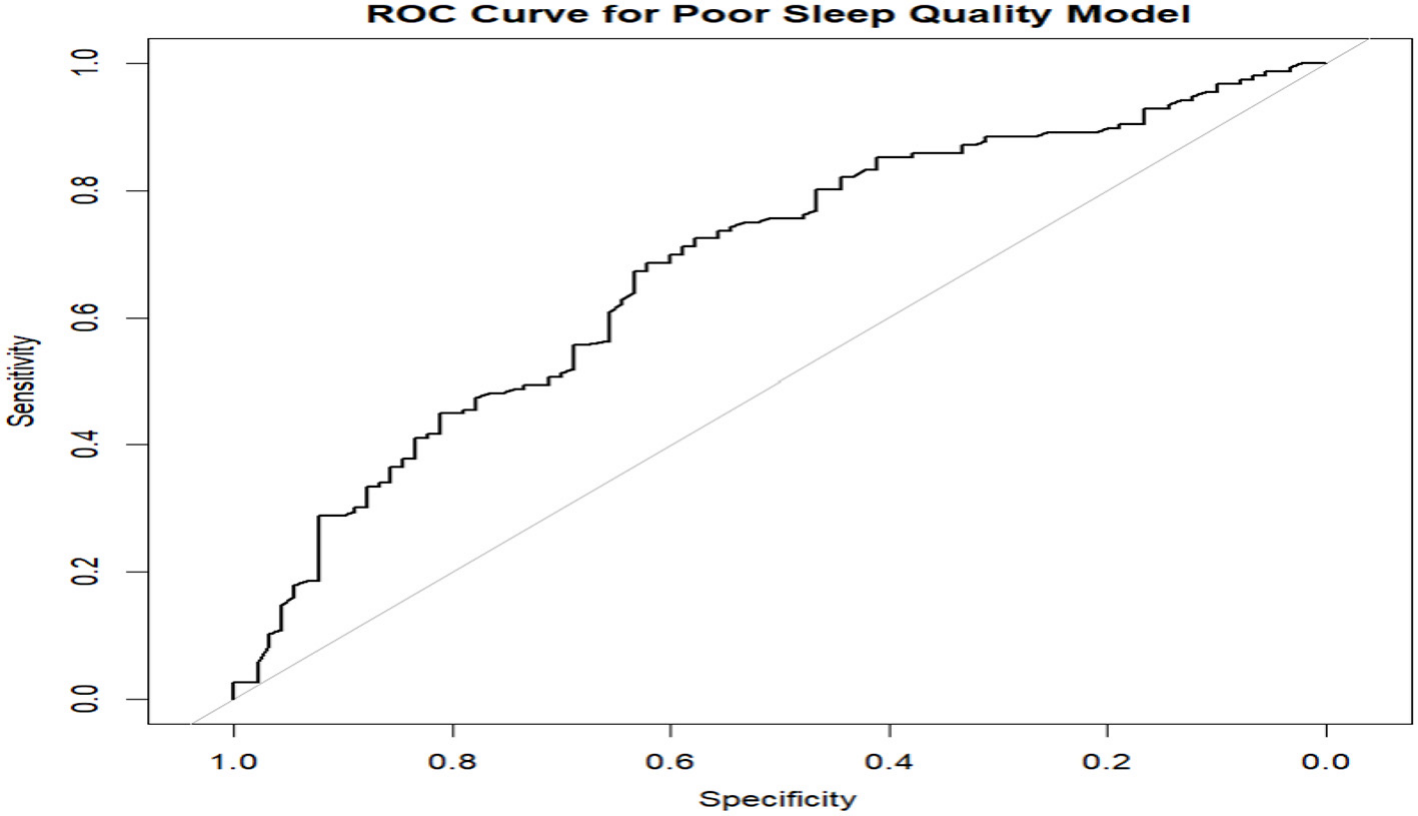
Receiver operating characteristic (ROC) curve for the logistic regression model predicting poor sleep quality (PSQI > 5).

The discriminative performance of the logistic regression model was evaluated using ROC curve analysis, which indicated acceptable model performance (AUC = 0.72, 95% CI: 0.66–0.78; Figure 1). Two-way ANOVA results for PSQI components and total score according to sex and sport type are presented in Table 4.

**Table 4:** Two-way ANOVA results for PSQI components and total score by sex and sport type.

Variable	Effect	*F* (1,494)	*p*	*ηp* ^2^
Subjective sleep quality	Sex	32.4	< 0.001	0.062
	Sport type	28.7	< 0.001	0.055
	Sex × Sport type	4.9	0.027	0.010
Sleep latency	Sex	41.8	< 0.001	0.078
	Sport type	19.6	< 0.001	0.038
	Sex × Sport type	1.8	0.176	0.004
Sleep duration	Sex	9.7	0.002	0.019
	Sport type	2.1	0.148	0.004
	Sex × Sport type	0.9	0.341	0.002
Habitual sleep efficiency	Sex	11.4	< 0.001	0.022
	Sport type	36.2	< 0.001	0.068
	Sex × Sport type	6.3	0.012	0.013
Sleep disturbance	Sex	27.9	< 0.001	0.054
	Sport type	22.5	< 0.001	0.044
	Sex × Sport type	2.6	0.107	0.005
Use of sleep medication	Sex	7.1	0.008	0.014
	Sport type	18.3	< 0.001	0.036
	Sex × Sport type	3.9	0.049	0.008
Daytime dysfunction	Sex	45.6	< 0.001	0.085
	Sport type	16.8	< 0.001	0.033
	Sex × Sport type	5.4	0.021	0.011
PSQI total score	Sex	52.3	< 0.001	0.096
	Sport type	40.1	< 0.001	0.075
	Sex × Sport type	7.2	0.008	0.015

PSQI, Pittsburgh sleep quality index. Partial eta squared (η*p*^2^) is reported as a measure of effect size.

**Figure 2:**
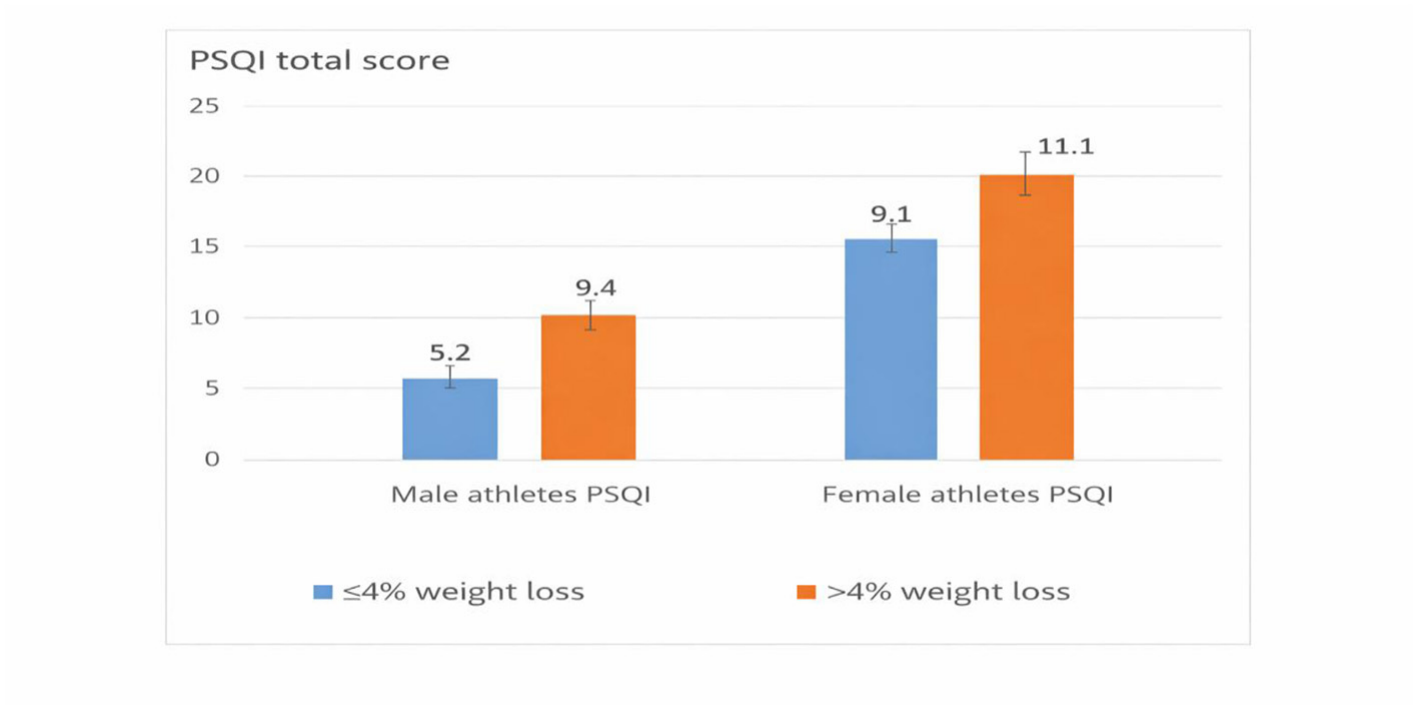
Mean PSQI total scores among Olympic combat sport athletes stratified by sex and weight loss level ( ≤ 4% vs. >4%).

Female athletes exhibited higher mean PSQI total scores compared to male athletes, whereas athletes reporting >4% weight loss showed higher PSQI scores than those reporting ≤ 4% weight loss (Figure 2).

**Figure 3:**
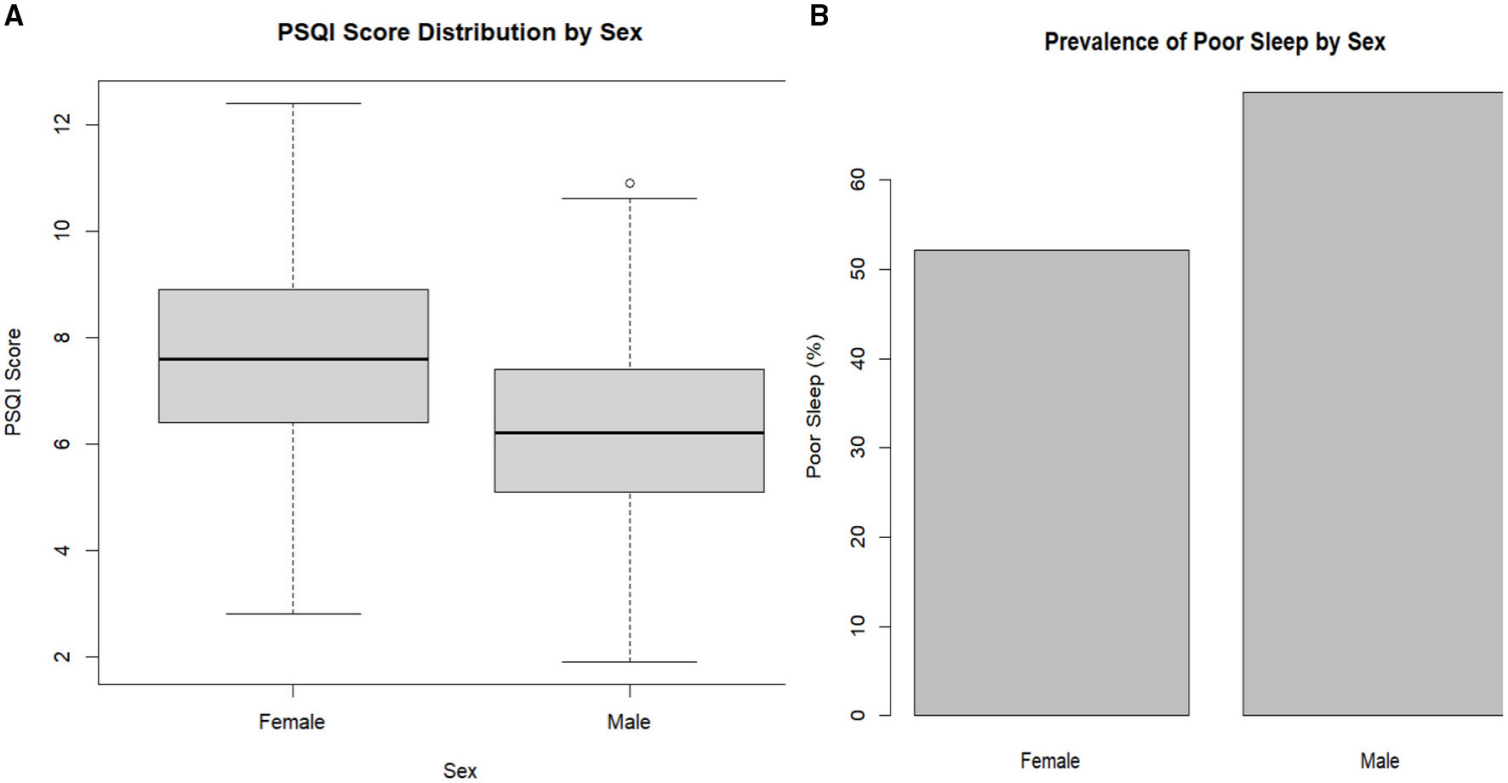
Sex differences in sleep quality. **(A)** Distribution of PSQI scores by sex. **(B)** Prevalence of poor sleep quality (PSQI > 5) by sex.

Female athletes exhibited higher PSQI total scores compared to male athletes, whereas male athletes demonstrated a higher prevalence of poor sleep quality (PSQI > 5), reflecting differences between continuous and categorical sleep outcomes (Figure 3).

Pairwise comparisons of PSQI components by sport type among female athletes are presented in Table 5. Significant differences were observed for subjective sleep quality, habitual sleep efficiency, sleep disturbance, daytime dysfunction, and the total PSQI score. In all significant comparisons, athletes competing in Olympic combat sports exhibited poorer sleep outcomes compared to those in non-Olympic disciplines. Effect sizes ranged from small to large, with the largest effect observed for subjective sleep quality (Hedges *g* = 0.75). No significant differences were found for sleep latency, sleep duration, or sleep medication use.

**Table 5:** Pairwise comparisons of PSQI components and total score by sport type in female athletes.

Variable	Sport type	*N*	Mean	SD	*t*	*p*	Hedges *g*	95% CI
Subjective sleep quality	Non-Olympic combat sports	128	0.89	0.72	2.74	0.003	0.75	0.50–1.00
	Olympic combat sports	110	1.98	0.81				
Sleep latency	Non-Olympic combat sports	128	2.10	0.66	0.99	0.396	−0.06	−0.32–0.20
	Olympic combat sports	110	2.06	0.76				
Sleep duration	Non-Olympic combat sports	128	0.89	0.63	0.95	0.428	−0.11	−0.37–0.15
	Olympic combat sports	110	0.82	0.62				
Habitual sleep efficiency	Non-Olympic combat sports	128	0.19	0.33	4.60	< 0.001	0.60	0.35–0.85
	Olympic combat sports	110	0.53	0.43				
Sleep disturbance	Non-Olympic combat sports	128	1.60	0.64	2.18	0.004	0.34	0.08–0.60
	Olympic combat sports	110	1.80	0.53				
Sleep medication use	Non-Olympic combat sports	128	0.24	0.34	0.83	0.542	0.20	−0.05–0.45
	Olympic combat sports	110	0.12	0.24				
Daytime dysfunction	Non-Olympic combat sports	128	1.37	0.61	2.46	0.003	0.68	0.42–0.94
	Olympic combat sports	110	1.78	0.60				
PSQI total score	Non-Olympic combat sports	128	7.5	3.0	6.70	0.001	0.55	0.30–0.80
	Olympic combat sports	110	9.0	3.1				

Values are presented as mean ± SD. *P*-values were adjusted using the Holm–Bonferroni method. Effect sizes are reported as Hedges' *g* with 95% confidence intervals.

Pairwise comparisons of PSQI components by sport type among male athletes are presented in Table 6. Significant differences were observed for subjective sleep quality, habitual sleep efficiency, sleep medication use, and the total PSQI score. In all significant comparisons, athletes competing in Olympic combat sports exhibited poorer sleep outcomes compared to those in non-Olympic disciplines. Effect sizes were in the small-to-moderate range. No significant differences were found for sleep latency, sleep duration, sleep disturbance, or daytime dysfunction.

**Table 6:** Pairwise comparisons of PSQI components and total score by sport type in male athletes.

Variable	Sport type	*N*	Mean	SD	*t*	*p*	Hedges *g*	95% CI
Subjective sleep quality	Non-Olympic combat sports	130	0.79	0.72	5.74	< 0.001	0.75	0.50–1.00
	Olympic combat sports	136	1.92	0.81				
Sleep latency	Non-Olympic combat sports	130	1.25	0.66	1.02	0.308	−0.03	−0.27–0.21
	Olympic combat sports	136	1.23	0.76				
Sleep duration	Non-Olympic combat sports	130	0.70	0.66	0.83	0.408	0.03	−0.21–0.27
	Olympic combat sports	136	0.72	0.69				
Habitual sleep efficiency	Non-Olympic combat sports	130	0.25	0.35	8.12	< 0.001	0.65	0.40–0.90
	Olympic combat sports	136	0.85	0.45				
Sleep disturbance	Non-Olympic combat sports	130	1.02	0.63	0.12	0.905	0.02	−0.22–0.26
	Olympic combat sports	136	1.03	0.54				
Sleep medication use	Non-Olympic combat sports	130	0.20	0.25	8.55	< 0.001	0.62	0.37–0.87
	Olympic combat sports	136	0.70	0.62				
Daytime dysfunction	Non-Olympic combat sports	130	0.96	0.60	1.66	0.098	0.13	−0.11–0.37
	Olympic combat sports	136	1.04	0.62				
PSQI total score	Non-Olympic combat sports	130	4.99	2.61	9.02	< 0.001	0.65	0.41–0.91
	Olympic combat sports	136	7.93	2.78				

*P*-values were adjusted using the Holm–Bonferroni method to control for family-wise error across multiple comparisons.

## 4 Discussion

The present findings suggest that sport-specific demands and rapid weight loss practices may be associated with sleep outcomes in combat sport athletes. This interpretation is consistent with previous research suggesting that both individual characteristics and sport-related demands are linked to sleep disturbances in athletes (Walsh et al., 2021). Rapid weight loss practices have also been associated with impairments in recovery and sleep quality in combat sport athletes (Lakicevic et al., 2024; Martínez-Aranda et al., 2023). In addition, athletes competing in Olympic combat sports are often exposed to structured competition schedules and weight-category demands, which may be related to a higher likelihood of sleep disturbances (Reale et al., 2017).

Despite the presence of multiple weight categories, the relatively narrow BMI distribution (23.1 kg/m^2^ ± 3.3 kg/m^2^; Table 1) may reflect the predominance of athletes in lower and middle weight classes. Detailed participant distribution according to generalized weight-category groupings is presented in Appendix Table A1. Additionally, BMI may not accurately represent body composition in athletes, as higher values can be influenced by increased muscle mass rather than adiposity. The present study examined sleep quality among athletes competing in Olympic and non-Olympic combat sports, with particular attention to sex differences and rapid weight loss practices. A key finding is the divergence between prevalence-based and continuous indicators of sleep quality. While male athletes demonstrated a higher prevalence of poor sleep quality (PSQI > 5), female athletes exhibited higher overall PSQI scores, reflecting greater symptom severity. This distinction highlights the difference between threshold-based and continuous measures: the PSQI cut-off identifies individuals exceeding a clinical threshold, whereas continuous scores capture the overall burden of sleep-related symptoms. Accordingly, male athletes may be more likely to exceed the threshold, while female athletes may experience greater symptom severity without crossing the cut-off. These findings suggest that prevalence and severity represent complementary but distinct dimensions of sleep quality in athlete populations (Mollayeva et al., 2016).

Previous research indicates that training intensity, exercise timing, and recovery demands are important factors associated with sleep patterns, with high-intensity and late-evening training associated with poorer sleep outcomes (Messman et al., 2024; Owoeye et al., 2024; Patel et al., 2024). In addition, insufficient recovery and overtraining have been linked to disruptions in circadian rhythms and reduced sleep quality (West, 2018; Stutz et al., 2019). In line with these findings, the higher PSQI scores observed in Olympic combat sport athletes and in those reporting greater weight loss may be related to sleep regulation difficulties associated with increased physiological load and recovery demands (Owoeye et al., 2024; Messman et al., 2024). Although the impact of sleep disturbances on athletic performance is well recognized, previous research has also reported variability in sleep quality across physically active populations, influenced by factors such as sample characteristics, activity level, and timing of assessment (Gohari et al., 2024; Saygili et al., 2011; Mesquita and Reimão, 2010; Güney and Imamoglu, 2022). Consistent with this variability, the present findings suggest that sex-related differences may represent an important factor in sleep patterns among combat sport athletes.

Several factors have been reported to be associated with sleep quality, including individual characteristics, health status, sleep duration, and levels of physiological and psychological stress (Paruthi et al., 2016). Previous studies have also documented sex-related differences in sleep quality, with potential explanations including hormonal fluctuations, perceived stress, and psychosocial factors affecting sleep patterns in women (Güney and Imamoglu, 2022; Keshavarz and Ghalebandi, 2009; Laposky et al., 2008; Orzech et al., 2011). Sleep disturbances in athletes are also associated with training load, competitive pressure, and demanding schedules (Walsh et al., 2021), and sleep quality has been shown to vary according to sport type and level of competition (Kirschen et al., 2020; Simpson et al., 2017). Consistent with this, the present study showed significant differences in several dimensions of sleep quality according to sport type, with athletes in Olympic combat sports showing higher values in PSQI components compared with their non-Olympic counterparts. Importantly, these differences were primarily observed in components related to sleep continuity, efficiency, and daytime functioning, rather than sleep duration. This may indicate that sport-specific demands in Olympic combat sports, such as weight-category pressure and intensive pre-competition preparation, are related to the qualitative aspects of sleep.

In the present study, athletes reporting weight loss exceeding 4% demonstrated a higher likelihood of poor sleep quality compared with those reporting ≤ 4% weight loss (Table 3), consistent with previous findings linking greater weight loss to higher PSQI scores (Drid et al., 2023). Rapid weight loss has been associated with sleep quality and recovery processes, although its impact may vary depending on individual characteristics and weight management strategies (Türkyilmaz and Yarar, 2024). Some evidence also suggests that female athletes may be more sensitive to sleep disturbances during periods of weight loss due to physiological and psychosocial factors. In addition, recent studies emphasize that weight management strategies should not focus solely on nutrition and physical activity but also consider sleep regulation as a relevant component of performance and recovery (Martínez-Aranda et al., 2023; Lebron et al., 2024; Knowlden et al., 2024). The relationship between sleep patterns and weight loss may be bidirectional, as insufficient sleep may impair weight management, whereas better sleep quality is associated with more favorable performance outcomes (Hatia et al., 2024; Doherty et al., 2024). In addition, sleep deprivation has been linked to alterations in carbohydrate metabolism, appetite regulation, and energy balance, which may further complicate weight control (Gangitano et al., 2023). These findings suggest that weight management practices in combat sport athletes should be considered alongside sleep regulation, as sleep disturbances may be associated with both recovery and performance.

## 5 Conclusions

Sleep quality differed according to sex and sport type in combat sport athletes, with male athletes showing a higher likelihood of poor sleep quality and female athletes demonstrating greater severity of sleep-related problems. Athletes competing in Olympic combat sports showed higher values in several sleep-related parameters compared with those in non-Olympic disciplines, and rapid weight loss was associated with a higher likelihood of poor sleep quality.

These findings suggest that sleep disturbances are common in combat sport athletes and may be related to sport-specific demands and rapid weight loss practices. Considering sleep regulation alongside training load and weight management may be relevant for supporting athlete health and performance. Future research should further examine these relationships using longitudinal designs and objective sleep measures.

### 5.1 Limitations and strengths of the study

The study was conducted with athletes recruited from licensed sports clubs and national-level training camps. Although all participants had recent national and/or international competition experience, potential differences in recruitment settings between Olympic and non-Olympic combat sport athletes may have introduced selection bias. Therefore, group comparisons should be interpreted with caution, as unmeasured differences in competitive level, training environment, or athlete support structures cannot be fully excluded. Importantly, objective indicators of competitive level (e.g., national rankings or competition results) were not collected, which limits the ability to fully determine whether the observed differences are attributable to sport type or underlying differences in athlete performance level.

In addition, the cross-sectional design of the study does not allow causal interpretation of the observed associations. Data related to sleep quality and weight loss were collected through self-report, which may introduce response bias. Furthermore, rapid weight loss was assessed based on short-term pre-competition weight changes and was not objectively verified, whereas sleep quality was evaluated over the previous month using the PSQI, which may result in a temporal mismatch between variables. Accordingly, findings related to rapid weight loss should be interpreted with caution. In addition, the absence of information on weight class limits the ability to account for weight-category–specific demands. The relatively narrow BMI distribution may also indicate that athletes from lower and middle weight categories were more represented in the sample, which should be considered when interpreting the generalizability of the findings. The lack of objective sleep assessment methods such as actigraphy or polysomnography represents another limitation.

Despite these limitations, the study includes a relatively large sample of combat sport athletes and provides a direct comparison between Olympic and non-Olympic disciplines. In addition, sleep quality was evaluated using both categorical and continuous indicators, allowing for the identification of differences between prevalence and symptom severity. Future research is recommended to include multi-center samples and objective sleep assessment methods.

### 5.2 Practical implications and suggestions

The findings of the present study highlight the importance of monitoring sleep quality in combat sport athletes, particularly during periods involving rapid weight loss and intensive training demands. Coaches and sports practitioners may benefit from incorporating sleep monitoring into routine training and recovery programs to better support athlete health and performance, such as using sleep diaries, wearable sleep tracking devices, or regular screening of sleep quality (e.g., PSQI).

Given the higher likelihood of poor sleep quality observed among athletes reporting weight loss greater than 4%, greater attention to sleep hygiene and recovery strategies during pre-competition weight management periods may be beneficial. In addition, the sex-related differences observed in sleep outcomes suggest that individualized recovery and sleep management strategies may help optimize sleep and overall athlete well-being. However, it should be noted that rapid weight loss was assessed retrospectively using self-reported data, which may introduce recall bias. In addition, the study did not differentiate between specific weight-cutting strategies (e.g., gradual dietary restriction vs. acute dehydration), and sleep quality was evaluated over the previous month using the PSQI, which may result in a temporal mismatch between variables. Therefore, practical implications related to rapid weight loss should be interpreted with caution.

### 5.3 Future research

Future studies should examine sleep quality in combat sport athletes using larger and multi-center samples to improve generalizability. Longitudinal research designs may help clarify the temporal relationships between rapid weight loss, training load, and sleep quality. In addition, the use of objective sleep assessment methods such as actigraphy or polysomnography may provide a more comprehensive understanding of sleep patterns in combat sport athletes.

## Data Availability

The raw data supporting the conclusions of this article will be made available by the authors, without undue reservation.

## Appendix

**Table A1:** Distribution of participants according to generalized weight-category groupings.

Weight-category grouping	Generalized weight range	*n*	%
Lower weight classes	≤ 66 kg	212	42.6
Middle weight classes	67–84 kg	198	39.8
Upper weight classes	≥85 kg	88	17.6
Total		498	100
